# Supplementary material for: Origin and Potential Expansion of the Invasive Longan Lanternfly, Pyrops candelaria (Hemiptera: Fulgoridae) in Taiwan
Source: Biology (Basel). 2021 Jul 17;10(7):678. doi: 10.3390/biology10070678 (PMC8301348; doi:10.3390/biology10070678)
Supplement: Supplementary file 1 [file biology-10-00678-s001.zip › Table S1.pdf]

**Table S1.** Collecting information for the samples of longan lanternfly used in the study and GenBank accession numbers.

| Sample code | Location | Date | Collector | GenBank accession No. for COI sequences | GenBank accession No. for ND2 sequences |
|---|---|---|---|---|---|
| CGD1 | Xianhu Botanical garden, Shenzhen, Guangdong, China | IX-07-2011 | Y.-Y. Li | KM244702 | KM244702 |
| CFJ1 | Fujian, China | - | - | FJ006724 | FJ006724 |
| CHN1 | Hainan, China | V-07-2020 | D.-Z. Qin | MZ350301 | MZ358925 |
| CHN2 | Hainan, China | V-07-2020 | D.-Z. Qin | MZ350302 | MZ358926 |
| CHN3 | Hainan, China | V-07-2020 | D.-Z. Qin | MZ350303 | MZ358927 |
| CHN4 | Hainan, China | V-07-2020 | D.-Z. Qin | MZ350304 | MZ358928 |
| CHN5 | Hainan, China | V-07-2020 | D.-Z. Qin | MZ350305 | MZ358929 |
| CHK1 | Hong Kong | X-22-2019 | C.-M. Leong | MZ350306 | MZ358930 |
| CHK2 | Hong Kong | XI-11-2019 | C.-M. Leong | MZ350307 | MZ358931 |
| CHK3 | Hong Kong | XI-11-2019 | C.-M. Leong | MZ350308 | MZ358932 |
| CMC1 | Macau | IX-09-2018 | C.-M. Leong | MZ350309 | MZ358933 |
| TKM1 | Kinmen, Taiwan | VII-12-2001 | H.-T. Shih | MZ350310 | MZ358934 |
| TKM2 | Kinmen, Taiwan | VII-12-2001 | H.-T. Shih | MZ350311 | MZ358935 |
| TKM3 | Kinmen, Taiwan | VII-12-2001 | H.-T. Shih | MZ350312 | MZ358936 |
| TKM4 | Lieyu, Kinmen, Taiwan | VIII-29-2018 | B.-T. Chen | MZ350313 | MZ358937 |
| TKM5 | Lieyu, Kinmen, Taiwan | VIII-29-2018 | B.-T. Chen | MZ350314 | MZ358938 |
| TKM6 | Lieyu, Kinmen, Taiwan | VIII-29-2018 | B.-T. Chen | MZ350315 | MZ358939 |
| TKM7 | Lieyu, Kinmen, Taiwan | VIII-29-2018 | B.-T. Chen | MZ350316 | MZ358940 |
| TKM8 | Lieyu, Kinmen, Taiwan | VIII-29-2018 | B.-T. Chen | MZ350317 | MZ358941 |
| TKM9 | Lieyu, Kinmen, Taiwan | VIII-29-2018 | B.-T. Chen | MZ350318 | MZ358942 |

| | | | | | |
|---|---|---|---|---|---|
| TKM10 | Lieyu, Kinmen, Taiwan | VIII-29-2018 | B.-T. Chen | MZ350319 | MZ358942 |
| TKM11 | Lieyu, Kinmen, Taiwan | VIII-29-2018 | B.-T. Chen | MZ350320 | MZ358944 |
| TKM12 | Lieyu, Kinmen, Taiwan | VIII-29-2018 | B.-T. Chen | MZ350321 | MZ358945 |
| TKM13 | Kinmen, Taiwan | IX-02-2020 | Y.-S. Lin | MZ350322 | MZ358946 |
| TKM14 | Kinmen, Taiwan | IX-02-2020 | Y.-S. Lin | MZ350323 | MZ358947 |
| TKM15 | Kinmen, Taiwan | IX-02-2020 | Y.-S. Lin | MZ350324 | MZ358948 |
| TKM16 | Kinmen, Taiwan | IX-02-2020 | Y.-S. Lin | MZ350325 | MZ358949 |
| TKM17 | Kinmen, Taiwan | IX-03-2020 | Y.-S. Lin | MZ350326 | MZ358950 |
| TKM18 | Kinmen, Taiwan | IX-03-2020 | Y.-S. Lin | MZ350327 | MZ358951 |
| TKM19 | Lieyu, Kinmen, Taiwan | IX-03-2020 | Y.-S. Lin | MZ350328 | MZ358952 |
| TKM20 | Lieyu, Kinmen, Taiwan | IX-03-2020 | Y.-S. Lin | MZ350329 | MZ358953 |
| TMA1 | Matsu, Taiwan | XI-23-2018 | D.-C. Chen | MZ350330 | MZ358954 |
| TBL1 | Bali, New Taipei City, Taiwan | VIII-27-2018 | C.-Y. Jiang | MZ350331 | MZ358955 |
| TBL2 | Bali, New Taipei City, Taiwan | VIII-27-2018 | C.-Y. Jiang | MZ350332 | MZ358956 |
| TBL3 | Bali, New Taipei City, Taiwan | VIII-27-2018 | C.-Y. Jiang | MZ350333 | MZ358957 |
| TBL4 | Bali, New Taipei City, Taiwan | VIII-27-2018 | C.-Y. Jiang | MZ350334 | MZ358958 |
| TBL5 | Bali, New Taipei City, Taiwan | VIII-27-2018 | C.-Y. Jiang | MZ350335 | MZ358959 |
| TBL6 | Bali, New Taipei City, Taiwan | VIII-27-2018 | C.-Y. Jiang | MZ350336 | MZ358960 |
| TBL7 | Bali, New Taipei City, Taiwan | VIII-27-2018 | C.-Y. Jiang | MZ350337 | MZ358961 |
| TBL8 | Bali, New Taipei City, Taiwan | VIII-27-2018 | C.-Y. Jiang | MZ350338 | MZ358962 |
| TBL9 | Bali, New Taipei City, Taiwan | VIII-27-2018 | C.-Y. Jiang | MZ350339 | MZ358963 |
| TBL10 | Bali, New Taipei City, Taiwan | IV-10-2019 | Y.-S. Lin | MZ350340 | MZ358964 |
| TBL11 | Bali, New Taipei City, Taiwan | IV-10-2019 | Y.-S. Lin | MZ350341 | MZ358965 |
| TBL12 | Bali, New Taipei City, Taiwan | IV-10-2019 | Y.-S. Lin | MZ350342 | MZ358966 |

| | | | | | |
|---|---|---|---|---|---|
| TBL13 | Bali, New Taipei City, Taiwan | IV-10-2019 | Y.-S. Lin | MZ350343 | MZ358967 |
| TBL14 | Bali, New Taipei City, Taiwan | IV-10-2019 | Y.-S. Lin | MZ350344 | MZ358968 |
| TBL15 | Bali, New Taipei City, Taiwan | IV-10-2019 | Y.-S. Lin | MZ350345 | MZ358969 |
| TBL16 | Bali, New Taipei City, Taiwan | IV-10-2019 | Y.-S. Lin | MZ350346 | MZ358970 |
| TBL17 | Bali, New Taipei City, Taiwan | VIII-22-2019 | Y.-S. Lin | MZ350347 | MZ358971 |
| TBL18 | Bali, New Taipei City, Taiwan | VIII-22-2019 | Y.-S. Lin | MZ350348 | MZ358972 |
| TBL19 | Bali, New Taipei City, Taiwan | VIII-29-2019 | Y.-S. Lin | MZ350349 | MZ358973 |
| TBL20 | Bali, New Taipei City, Taiwan | VIII-29-2019 | Y.-S. Lin | MZ350350 | MZ358974 |
| TBL21 | Bali, New Taipei City, Taiwan | VIII-29-2019 | Y.-S. Lin | MZ350351 | MZ358975 |
| TBL22 | Bali, New Taipei City, Taiwan | VIII-29-2019 | Y.-S. Lin | MZ350352 | MZ358976 |
| TBL23 | Bali, New Taipei City, Taiwan | VIII-29-2019 | Y.-S. Lin | MZ350353 | MZ358977 |
| TBL24 | Bali, New Taipei City, Taiwan | VIII-29-2019 | Y.-S. Lin | MZ350354 | MZ358978 |
| TBL25 | Bali, New Taipei City, Taiwan | VIII-29-2019 | Y.-S. Lin | MZ350355 | MZ358979 |
| TBL26 | Bali, New Taipei City, Taiwan | VIII-29-2019 | Y.-S. Lin | MZ350356 | MZ358980 |
| TBL27 | Bali, New Taipei City, Taiwan | VIII-29-2019 | Y.-S. Lin | MZ350357 | MZ358981 |
| TBT1 | Beitou, Taipei, Taiwan | IX-21-2018 | BAPHIQ | MZ350358 | MZ358982 |
| TBT2 | Beitou, Taipei, Taiwan | IX-21-2018 | BAPHIQ | MZ350359 | MZ358983 |
| TBT3 | Beitou, Taipei, Taiwan | IX-21-2018 | BAPHIQ | MZ350360 | MZ358984 |
| TBT4 | Beitou, Taipei, Taiwan | VIII-28-2019 | Y.-S. Lin | MZ350361 | MZ358985 |
| TBT5 | Beitou, Taipei, Taiwan | VIII-28-2019 | Y.-S. Lin | MZ350362 | MZ358986 |
| TBT6 | Beitou, Taipei, Taiwan | VIII-28-2019 | Y.-S. Lin | MZ350363 | MZ358987 |
| TBT7 | Guizikeng, Beitou, Taipei, Taiwan | VII-16-2019 | Y.-S. Lin | MZ350364 | MZ358988 |
| TBT8 | Guizikeng, Beitou, Taipei, Taiwan | VIII-28-2019 | Y.-S. Lin | MZ350365 | MZ358989 |

| | | | | | |
|---|---|---|---|---|---|
| TBT9 | Guizikeng, Beitou, Taipei, Taiwan | VIII-28-2019 | Y.-S. Lin | MZ350366 | MZ358990 |
| TBT10 | Guizikeng, Beitou, Taipei, Taiwan | VIII-28-2019 | Y.-S. Lin | MZ350367 | MZ358991 |
| TBT11 | Guizikeng, Beitou, Taipei, Taiwan | VIII-28-2019 | Y.-S. Lin | MZ350368 | MZ358992 |
| TBT12 | Guizikeng, Beitou, Taipei, Taiwan | VIII-28-2019 | Y.-S. Lin | MZ350369 | MZ358993 |
| TBT13 | Guizikeng, Beitou, Taipei, Taiwan | VIII-28-2019 | Y.-S. Lin | MZ350370 | MZ358994 |
| TBT14 | Guizikeng, Beitou, Taipei, Taiwan | X-05-2019 | S.-H. Lin | MZ350371 | MZ358995 |
| TBT15 | Guizikeng, Beitou, Taipei, Taiwan | X-05-2019 | S.-H. Lin | MZ350372 | MZ358996 |
| TBT16 | Guizikeng, Beitou, Taipei, Taiwan | X-05-2019 | S.-H. Lin | MZ350373 | MZ358997 |
| TBT17 | Guizikeng, Beitou, Taipei, Taiwan | X-05-2019 | S.-H. Lin | MZ350374 | MZ358998 |
| TBT18 | Guizikeng, Beitou, Taipei, Taiwan | X-05-2019 | S.-H. Lin | MZ350375 | MZ358999 |
| TBT19 | Beitou, Taipei, Taiwan | X-09-2019 | Y.-S. Lin | MZ350376 | MZ359000 |
| TBT20 | Beitou, Taipei, Taiwan | X-09-2019 | Y.-S. Lin | MZ350377 | MZ359001 |
| TBT21 | Beitou, Taipei, Taiwan | X-26-2019 | S.-H. Lin | MZ350378 | MZ359002 |
| TBT22 | Beitou, Taipei, Taiwan | IV-20-2020 | Y.-S. Lin | MZ350379 | MZ359003 |
| TGM1 | Guanyin Moutain, New Taipei City, Taiwan | X-23-2018 | C.-Y. Jiang | MZ350380 | MZ359004 |
| TGM2 | Guanyin Moutain, New Taipei | X-23-2018 | C.-Y. Jiang | MZ350381 | MZ359005 |

| | | | | | |
|---|---|---|---|---|---|
| | City, Taiwan | | | | |
| TGM3 | Guanyin Moutain, New Taipei City, Taiwan | X-23-2018 | C.-Y. Jiang | MZ350382 | MZ359006 |
| TGM4 | Guanyin Moutain, New Taipei City, Taiwan | X-23-2018 | C.-Y. Jiang | MZ350383 | MZ359007 |
| TGM5 | Guanyin Moutain, New Taipei City, Taiwan | X-23-2018 | C.-Y. Jiang | MZ350384 | MZ359008 |
| TGM6 | Guanyin Moutain, New Taipei City, Taiwan | X-23-2018 | C.-Y. Jiang | MZ350385 | MZ359009 |
| TGM7 | Guanyin Moutain, New Taipei City, Taiwan | X-23-2018 | C.-Y. Jiang | MZ350386 | MZ359010 |
| TGM8 | Guanyin Moutain, New Taipei City, Taiwan | X-23-2018 | C.-Y. Jiang | MZ350387 | MZ359011 |
| TGM9 | Guanyin Moutain, New Taipei City, Taiwan | X-23-2018 | C.-Y. Jiang | MZ350388 | MZ359012 |
| TGM10 | Guanyin Moutain, New Taipei City, Taiwan | X-23-2018 | C.-Y. Jiang | MZ350389 | MZ359013 |
| TGM11 | Guanyin Moutain, New Taipei City, Taiwan | X-23-2018 | C.-Y. Jiang | MZ350390 | MZ359014 |
| TGM12 | Guanyin Moutain, New Taipei City, Taiwan | X-23-2018 | C.-Y. Jiang | MZ350391 | MZ359015 |
| TGM13 | Guanyin Moutain, New Taipei City, Taiwan | X-23-2018 | C.-Y. Jiang | MZ350392 | MZ359016 |
| TGM14 | Guanyin Moutain, New Taipei City, Taiwan | X-23-2018 | C.-Y. Jiang | MZ350393 | MZ359017 |
| TGM15 | Guanyin Moutain, New Taipei City, Taiwan | X-23-2018 | C.-Y. Jiang | MZ350394 | MZ359018 |
| TLK1 | Linkou, Taipei, Taiwan | VIII-24-2020 | Y.-S. Lin | MZ350395 | MZ359019 |

| | | | | | |
|---|---|---|---|---|---|
| TLK2 | Linkou, Taipei, Taiwan | VIII-24-2020 | Y.-S. Lin | MZ350396 | MZ359020 |
| TLK3 | Linkou, Taipei, Taiwan | VIII-24-2020 | Y.-S. Lin | MZ350397 | MZ359021 |
| TLK4 | Linkou, Taipei, Taiwan | VIII-24-2020 | Y.-S. Lin | MZ350398 | MZ359022 |
| TLK5 | Linkou, Taipei, Taiwan | VIII-24-2020 | Y.-S. Lin | MZ350399 | MZ359023 |
| TLK6 | Linkou, Taipei, Taiwan | VIII-24-2020 | Y.-S. Lin | MZ350400 | MZ359024 |
| TLK7 | Linkou, Taipei, Taiwan | VIII-24-2020 | Y.-S. Lin | MZ350401 | MZ359025 |
| TLK8 | Linkou, Taipei, Taiwan | VIII-24-2020 | Y.-S. Lin | MZ350402 | MZ359026 |
| TLK9 | Linkou, Taipei, Taiwan | VIII-24-2020 | Y.-S. Lin | MZ350403 | MZ359027 |
| TLK10 | Linkou, Taipei, Taiwan | VIII-24-2020 | Y.-S. Lin | MZ350404 | MZ359028 |
| TLK11 | Linkou, Taipei, Taiwan | VIII-24-2020 | Y.-S. Lin | MZ350405 | MZ359029 |
| TLK12 | Linkou, Taipei, Taiwan | VIII-24-2020 | Y.-S. Lin | MZ350406 | MZ359030 |
| TLK13 | Linkou, Taipei, Taiwan | VIII-24-2020 | Y.-S. Lin | MZ350407 | MZ359031 |
| TLK14 | Linkou, Taipei, Taiwan | VIII-24-2020 | Y.-S. Lin | MZ350408 | MZ359032 |
| TLK15 | Linkou, Taipei, Taiwan | VIII-24-2020 | Y.-S. Lin | MZ350409 | MZ359033 |
| TLK16 | Linkou, Taipei, Taiwan | VIII-24-2020 | Y.-S. Lin | MZ350410 | MZ359034 |
| TLK17 | Linkou, Taipei, Taiwan | VIII-24-2020 | Y.-S. Lin | MZ350411 | MZ359035 |
| TLK18 | Linkou, Taipei, Taiwan | VIII-24-2020 | Y.-S. Lin | MZ350412 | MZ359036 |
| TLK19 | Linkou, Taipei, Taiwan | VIII-24-2020 | Y.-S. Lin | MZ350413 | MZ359037 |
| TLK20 | Linkou, Taipei, Taiwan | VIII-24-2020 | Y.-S. Lin | MZ350414 | MZ359038 |
| TWG1 | Wugu, New Taipei City, Taiwan | XI-16-2018 | C.-Y. Jiang | MZ350415 | MZ359039 |
| TWG2 | Wugu, New Taipei City, Taiwan | XI-16-2018 | C.-Y. Jiang | MZ350416 | MZ359040 |
| TWG3 | Wugu, New Taipei City, Taiwan | XI-16-2018 | C.-Y. Jiang | MZ350417 | MZ359041 |
| TWG4 | Wugu, New Taipei City, Taiwan | XI-16-2018 | C.-Y. Jiang | MZ350418 | MZ359042 |
| TWG5 | Wugu, New Taipei City, Taiwan | XI-16-2018 | C.-Y. Jiang | MZ350419 | MZ359043 |

| | | | | | |
|---|---|---|---|---|---|
| TWG6 | Wugu, New Taipei City, Taiwan | XI-16-2018 | C.-Y. Jiang | MZ350420 | MZ359044 |
| TWG7 | Wugu, New Taipei City, Taiwan | XI-16-2018 | C.-Y. Jiang | MZ350421 | MZ359045 |
| TWG8 | Wugu, New Taipei City, Taiwan | XI-16-2018 | C.-Y. Jiang | MZ350422 | MZ359046 |
| TWG9 | Wugu, New Taipei City, Taiwan | XI-16-2018 | C.-Y. Jiang | MZ350423 | MZ359047 |
| TWG10 | Wugu, New Taipei City, Taiwan | XI-16-2018 | C.-Y. Jiang | MZ350424 | MZ359048 |
| TWG11 | Wugu, New Taipei City, Taiwan | VIII-29-2019 | Y.-S. Lin | MZ350425 | MZ359049 |
| TWG12 | Wugu, New Taipei City, Taiwan | VIII-29-2019 | Y.-S. Lin | MZ350426 | MZ359050 |
| TWG13 | Wugu, New Taipei City, Taiwan | VIII-29-2019 | Y.-S. Lin | MZ350427 | MZ359051 |
| TWG14 | Wugu, New Taipei City, Taiwan | VIII-29-2019 | Y.-S. Lin | MZ350428 | MZ359052 |
| TWG15 | Wugu, New Taipei City, Taiwan | VIII-29-2019 | Y.-S. Lin | MZ350429 | MZ359053 |
| TWG16 | Wugu, New Taipei City, Taiwan | VIII-29-2019 | Y.-S. Lin | MZ350430 | MZ359054 |
| TWG17 | Wugu, New Taipei City, Taiwan | VIII-29-2019 | Y.-S. Lin | MZ350431 | MZ359055 |
| TWG18 | Wugu, New Taipei City, Taiwan | VIII-29-2019 | Y.-S. Lin | MZ350432 | MZ359056 |
| TWG19 | Wugu, New Taipei City, Taiwan | VIII-29-2019 | Y.-S. Lin | MZ350433 | MZ359057 |
| TWG20 | Wugu, New Taipei City, Taiwan | XI-13-2019 | Y.-S. Lin | MZ350434 | MZ359058 |
| TZH1 | Zhonghe, Taipei, Taiwan | VIII-06-2019 | BAPHIQ | MZ350435 | MZ359059 |
| THAI1 | Chiang Mai, Thailand | III-05-2020 | K. Jiaranaisakul | MZ350436 | MZ359060 |
| THAI2 | Chiang Mai, Thailand | III-05-2020 | K. Jiaranaisakul | MZ350437 | MZ359061 |
| THAI3 | Chiang Mai, Thailand | III-05-2020 | K. Jiaranaisakul | MZ350438 | MZ359062 |
| THAI4 | Chiang Mai, Thailand | III-05-2020 | K. Jiaranaisakul | MZ350439 | MZ359063 |
| THAI5 | Soi Dao, Chantaburi, Thailand | II-16-2020 | K. Jiaranaisakul | MZ350440 | MZ359064 |
| THAI6 | Suan Phueng, Ratchaburi, Thailand | V-13-2020 | K. Jiaranaisakul | MZ350441 | MZ359065 |
| THAI7 | Suan Phueng, Ratchaburi, Thailand | X-18-2020 | K. Jiaranaisakul | MZ350442 | MZ359066 |

| | | | | | |
|---|---|---|---|---|---|
| THAI8 | Suan Phueng, Ratchaburi, Thailand | X-18-2020 | K. Jiaranaisakul | MZ350443 | MZ359067 |
| THAI9 | Suan Phueng, Ratchaburi, Thailand | X-18-2020 | K. Jiaranaisakul | MZ350444 | MZ359068 |
| THAI10 | Suan Phueng, Ratchaburi, Thailand | X-20-2020 | K. Jiaranaisakul | MZ350445 | MZ359069 |
| THAI11 | Suan Phueng, Ratchaburi, Thailand | X-20-2020 | K. Jiaranaisakul | MZ350446 | MZ359070 |
| THAI12 | Ratchasima, Thailand | - | K. Jiaranaisakul | MZ350447 | MZ359071 |
| THAI13 | Ratchasima, Thailand | - | K. Jiaranaisakul | MZ350448 | MZ359072 |
| THAI14 | Mae Sai, Chiang Rai, Thailand | X-2020 | K. Jiaranaisakul | MZ350449 | MZ359073 |
| THAI15 | Thailand | - | K. Jiaranaisakul | MZ350450 | MZ359074 |
| THAI16 | Thailand | - | K. Jiaranaisakul | MZ350451 | MZ359075 |
| THAI17 | Thailand | - | K. Jiaranaisakul | MZ350452 | MZ359076 |
| THAI18 | Thailand | - | K. Jiaranaisakul | MZ350453 | MZ359077 |
| THAI19 | Thailand | - | K. Jiaranaisakul | MZ350454 | MZ359078 |
| THAI20 | Thailand | - | K. Jiaranaisakul | MZ350455 | MZ359079 |
