# Supplementary material for: Origin and Potential Expansion of the Invasive Longan Lanternfly, Pyrops candelaria (Hemiptera: Fulgoridae) in Taiwan
Source: Biology (Basel). 2021 Jul 17;10(7):678. doi: 10.3390/biology10070678 (PMC8301348; doi:10.3390/biology10070678)
Supplement: Supplementary file 1 [file biology-10-00678-s001.zip › Table-S4.pdf]

Table S4. Pairwise genetic distances between different populations of *Pyrops candelaria* based on partial mitochondrial COI and ND2 sequences.

	TBL	TWG	TGM	TBT	TLK	TZH	TKM	TMA	CMC	CHK	CGD	CHN	CFJ	THAI
TBL														
TWG	0.0000													
TGM	0.0000	0.0000												
TBT	0.0000	0.0000	0.0000											
TLK	0.0000	0.0000	0.0000	0.0000										
TZH	0.0000	0.0000	0.0000	0.0000	0.0000									
TKM	0.0010	0.0010	0.0010	0.0010	0.0010	0.0010								
TMA	0.0025	0.0025	0.0025	0.0025	0.0025	0.0025	0.0027							
CMC	0.0008	0.0008	0.0008	0.0008	0.0008	0.0008	0.0010	0.0017						
CHK	0.0008	0.0008	0.0008	0.0008	0.0008	0.0008	0.0013	0.0022	0.0006					
CGD	0.0025	0.0025	0.0025	0.0025	0.0025	0.0025	0.0023	0.0033	0.0017	0.0022				
CHN	0.0033	0.0033	0.0033	0.0033	0.0033	0.0033	0.0041	0.0055	0.0038	0.0039	0.0055			
CFJ	0.0109	0.0109	0.0109	0.0109	0.0109	0.0109	0.0111	0.0117	0.010	0.0106	0.0117	0.0139		
THAI	0.0032	0.0032	0.0032	0.0032	0.0032	0.0032	0.0035	0.0041	0.0024	0.0030	0.0041	0.0062	0.0125	

TBL = Bali; TWG = Wugu; TGM = Guanyin Mountain; TBT = Beitou; TLK = Linkou; TZH = Zhonghe; TKM = Kinmen; TMA = Matsu; CMC = Macau; CHK = Hong Kong; CGD = Guangdong; CHN = Hainan; CFJ = Fujian; THAI = Thailand.
